# Congenital Esophageal Stenosis Presenting During Weaning in a 5-Month-Old Infant

**DOI:** 10.3390/children13050706

**Published:** 2026-05-21

**Authors:** Massimo Verdicchio, Cristina Bucci, Sara Isoldi, Laura Aurino, Maria Giovanna Puoti, Serena Marulo, Federica Riccitiello, Rossella Turco, Francesco Cirillo, Giovanni Di Nardo, Paolo Quitadamo

**Affiliations:** 1 Pediatric Department, University of Salerno, 84084 Fisciano, Italy; 2 Pediatric Gastroenterology and Endoscopy Unit, Department of Pediatric Specialties, Santobono Children’s Hospital, 80129 Naples, Italy; c.bucci@santobonopausilipon.it (C.B.); s.isoldi@santobonopausilipon.it (S.I.); l.aurino@santobonopausilipon.it (L.A.); m.puoti@santobonopausilipon.it (M.G.P.); s.marulo@santobonopausilipon.it (S.M.); r.turco@santobonopausilipon.it (R.T.); f.cirillo@santobonopausilipon.it (F.C.); g.dinardo@santobonopausilipon.it (G.D.N.); p.quitadamo@santobonopausilipon.it (P.Q.); 3 Department of Radiology, Santobono Children’s Hospital, 80129 Naples, Italy; f.riccitiello@santobonopausilipon.it

**Keywords:** congenital esophageal stenosis, dysphagia, infant, feeding difficulties, esophagography, pediatric gastroenterology

## Abstract

**Highlights:**

**What is the main finding?**

- Congenital esophageal stenosis may present with progressive dysphagia during weaning and often requires repeated endoscopic dilations.

**What is the implication of the main finding?**

- Early structural evaluation with contrast esophagography and endoscopy is essential in infants with persistent feeding difficulties resistant to anti-reflux therapy.

**Abstract:**

Background/Objectives: Congenital esophageal stenosis (CES) is a rare condition that may present with feeding difficulties in infancy, often becoming evident during weaning. We report a case of CES presenting with progressive dysphagia during the introduction of solid foods. Methods: Clinical evaluation, contrast esophagography, endoscopy, and magnetic resonance imaging were performed to characterize the stenosis and guide management. Results: Imaging revealed a fixed narrowing of the mid-distal esophagus with minimal passage of contrast and proximal dilatation. Endoscopy confirmed a non-traversable intrinsic stenosis with macroscopically normal mucosa. The patient underwent three endoscopic dilation sessions under general anesthesia using Savary-Gilliard bougies with progressive diameters (3–5 mm, 3–7 mm, and 7–11 mm). No major procedural complications occurred. At 2-month follow-up after the last dilation, feeding was appropriate for age, dysphagia had improved, and growth was regular. Conclusions: CES should be suspected in infants with feeding difficulties that worsen during weaning, particularly when symptoms are resistant to anti-reflux therapy. Endoscopic dilation can improve feeding tolerance, although repeated and staged sessions may be required. The role of adjunctive therapies such as topical budesonide remains uncertain.

## 1. Introduction

Congenital esophageal stenosis (CES) is a rare intrinsic narrowing of the esophageal lumen, with an estimated incidence of approximately 1 in 25,000–50,000 live births [1]. It typically presents in infancy or early childhood with regurgitation, vomiting, feeding difficulties, dysphagia, or food impaction, particularly after the introduction of more viscous or solid foods. CES is classically classified into three main subtypes: tracheobronchial remnants, fibromuscular hypertrophy, and membranous diaphragm, each with different therapeutic implications [1,2].

The clinical diagnosis may be challenging because early symptoms often overlap with more common pediatric conditions, including gastroesophageal reflux disease (GERD), swallowing dysfunction, and, less frequently, eosinophilic esophagitis (EoE) or extrinsic esophageal compression [2,3]. Partial or transient response to empirical anti-reflux treatment may further delay diagnostic evaluation.

Delayed recognition of CES may lead to persistent vomiting, feeding aversion, recurrent aspiration, food impaction, or impaired growth. In this context, worsening symptoms during weaning represent an important clinical clue [2,4].

For this reason, persistent or progressive symptoms despite medical therapy should prompt structural evaluation of the esophagus. This case is clinically relevant due to the early presentation during weaning and the need for repeated dilation sessions to achieve sustained clinical improvement.

### Case Presentation

A 5-month-old infant presented with recurrent regurgitation and vomiting since early life, only partially responsive to alginate-based therapy. Alginate-based therapy was used intermittently, typically for short periods of 10–15 days, with partial and transient benefit. Proton pump inhibitors were not administered. No history of choking, cyanosis, recurrent respiratory infections, or aspiration was reported. A single episode of food impaction was described. The infant was born at term at 38 weeks of gestation after an uneventful pregnancy and neonatal period. Birth weight was not available.

The patient was initially fed with mixed feeding (breast milk and formula), which was relatively well tolerated. Weaning was initiated at 5 months of age with semi-solid foods, including vegetable-based purees. Symptoms were more pronounced with semi-solid and solid foods, while liquid feeding was relatively better tolerated, with progressive dysphagia and slow swallowing. At presentation, growth parameters were within the normal range, with both weight and length at approximately the 50th percentile. Physical examination was unremarkable.

Routine laboratory investigations, including complete blood count, inflammatory markers, and basic biochemical tests, were within normal limits.

**Figure 1:**
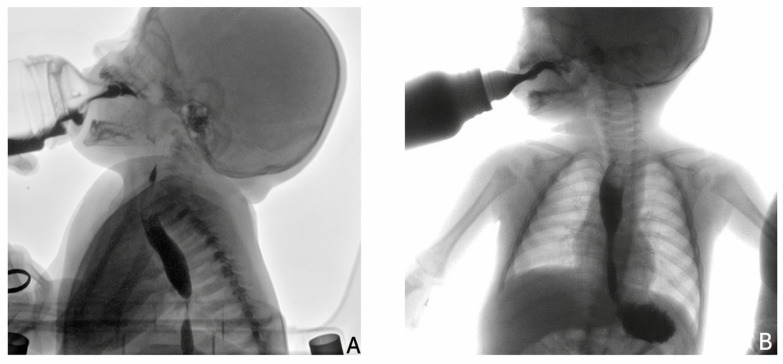
Contrast esophagography in a 5-month-old infant with congenital esophageal stenosis. (**A**) Lateral view showing marked narrowing of the mid-distal esophagus with mild proximal dilatation. (**B**) Anteroposterior view confirming reduced passage of contrast through the stenotic tract. The stenotic segment measured approximately 18 mm in length on MRI.

Contrast esophagography revealed a marked narrowing of the mid-distal esophagus, allowing only minimal passage of contrast, with mild proximal dilatation. The lateral projection (Figure 1A) demonstrated marked narrowing of the mid-distal esophagus with proximal dilatation, while the anteroposterior view (Figure 1B) confirmed reduced contrast passage through the stenotic segment. Mediastinal magnetic resonance imaging was performed to exclude extrinsic compression and associated mediastinal anomalies. MRI confirmed a segmental stenosis approximately 18 mm in length, without evidence of extrinsic compression or tracheobronchial remnants. Imaging findings were considered suggestive of a fibromuscular subtype of congenital esophageal stenosis [5].

Endoscopic evaluation identified a stenotic segment located 17 cm from the incisors that could not be traversed. The esophageal mucosa appeared macroscopically normal, with no evidence of rings, furrows, erosions, or ulceration suggestive of eosinophilic esophagitis. Biopsies were not obtained. Endoscopic images were not available for publication. The patient underwent three endoscopic dilation sessions under general anesthesia using Savary-Gilliard bougies. Progressive diameters were used in each session (3, 4, and 5 mm in the first session; 3, 5, and 7 mm in the second; 7, 9, and 11 mm in the third), with progressive improvement in dysphagia. No procedure-related complications, including perforation or significant bleeding, were observed.

A trial of viscous topical budesonide, administered by swallowing, was initiated empirically after repeated dilations, in the absence of endoscopic features suggestive of eosinophilic esophagitis. The rationale was to reduce potential subclinical mucosal inflammation and improve esophageal compliance. Its use was empirical and not intended as standard treatment for CES. At 2-month follow-up after the last dilation, during which daily budesonide therapy was continued, feeding was appropriate for age, dysphagia had improved, and growth was regular.

## 2. Discussion

Congenital esophageal stenosis (CES) is a rare but clinically significant cause of feeding difficulties in infancy [1,2], often becoming evident during the weaning period when the introduction of more viscous or solid foods unmasks an underlying structural abnormality. In early infancy, symptoms such as regurgitation and vomiting may overlap with more common conditions, including gastroesophageal reflux disease (GERD) and, less frequently, eosinophilic esophagitis (EoE), potentially leading to delayed diagnosis or inappropriate treatment [2,3]. In our case, gastroesophageal reflux disease was considered but was unlikely due to the persistence and progression of symptoms despite intermittent anti-reflux therapy. Eosinophilic esophagitis was considered less likely in the absence of typical endoscopic findings, although histological confirmation was not available. Extrinsic compression was excluded by magnetic resonance imaging.

In this context, the persistence or progression of symptoms despite anti-reflux therapy should raise suspicion of an underlying anatomical disorder. Our patient presented with early symptoms partially responsive to medical therapy, followed by progressive dysphagia during weaning, a pattern that is consistent with previously reported cases but often underrecognized in clinical practice.

Diagnosis of CES relies primarily on contrast esophagography and endoscopic evaluation [2,3]. Contrast studies are useful to define the site, length, and functional severity of the stenosis, typically showing a focal narrowing with proximal dilatation. Endoscopy provides direct visualization and allows assessment of the lumen patency, although the stenotic segment may not always be traversable, as observed in our case. Magnetic resonance imaging is not routinely required but may be helpful in selected cases to exclude extrinsic compression or associated mediastinal anomalies, particularly when imaging findings are atypical or when further anatomical characterization is needed.

From a pathophysiological perspective, CES is traditionally classified into three histological subtypes: tracheobronchial remnants, fibromuscular hypertrophy, and membranous diaphragm [1,4]. This classification is clinically relevant because it influences management strategies. In our case, magnetic resonance imaging findings were suggestive of a fibromuscular subtype, which is generally associated with a better response to endoscopic dilation compared to other forms such as tracheobronchial remnants. Histological confirmation was not available because surgical resection was not performed. Endoscopic dilation is generally effective in fibromuscular and membranous forms, whereas cases associated with tracheobronchial remnants may be more resistant and often require surgical resection. Therefore, management was guided by clinical and endoscopic response.

Endoscopic dilation remains the first-line therapeutic approach in most cases of CES. However, repeated sessions are frequently required to achieve and maintain adequate luminal patency. The need for multiple dilations in our patient is consistent with the literature, where gradual and staged dilation is often preferred to reduce the risk of complications while improving clinical outcomes [4,6]. Importantly, no major complications such as perforation or significant bleeding were observed in our case, supporting the safety of a cautious and progressive dilation strategy in experienced hands. Bougie dilation was selected as the preferred technique at our center based on operator experience, although balloon dilation represents a valid alternative approach described in the literature. In refractory or recurrent cases, alternative strategies such as temporary expandable stent placement may be considered, although their use in infants remains limited and should be carefully individualized.

The use of topical budesonide in this case deserves specific discussion. Topical corticosteroids have an established role in eosinophilic esophagitis and have also been explored in acquired or post-inflammatory esophageal strictures, including caustic injuries, post-dilation recurrence, and post-procedural stenosis [7,8]. In these settings, local steroid therapy has been shown to reduce inflammation, fibrosis, and the risk of restenosis, potentially decreasing the need for repeated dilations. 

However, these data cannot be directly extrapolated to congenital esophageal stenosis, where the underlying pathophysiology is primarily structural rather than inflammatory. In our patient, viscous budesonide was introduced empirically as an adjunctive therapy after repeated dilations, with the aim of reducing mucosal inflammation and improving esophageal compliance. Although clinical improvement was observed, it is not possible to determine the relative contribution of budesonide compared to repeated dilations and dietary management [9].

Therefore, topical budesonide should not be considered standard therapy for CES, and its use should remain individualized until further evidence becomes available.

Another relevant aspect is the potential for delayed diagnosis. CES is often overlooked in the early stages due to nonspecific symptoms and partial response to empirical therapies. This may result in prolonged feeding difficulties, feeding aversion, and, in more severe cases, failure to thrive or recurrent aspiration. Our case reinforces the importance of considering CES in infants with persistent or progressive symptoms, particularly when clinical deterioration occurs during weaning.

Previously reported cases have similarly described symptom worsening during weaning and the frequent need for repeated dilation procedures, although clinical presentation and therapeutic response may vary according to the underlying subtype.

Further prospective studies are needed to better define the role of adjunctive therapies and optimize long-term outcomes in congenital esophageal stenosis.

This case is noteworthy for the early clinical presentation during the weaning period and the need for repeated dilation sessions, highlighting the importance of early recognition and close follow-up.

## 3. Conclusions

Congenital esophageal stenosis should be considered in infants presenting with persistent vomiting, dysphagia, or feeding difficulties, particularly when symptoms worsen during the weaning period or show incomplete or transient response to anti-reflux therapy. In this setting, the progression of symptoms with the introduction of more viscous or solid foods should raise early suspicion of an underlying structural abnormality.

This case emphasizes the importance of a structured diagnostic approach based on contrast esophagography and endoscopic evaluation, which remain the cornerstone for identifying and characterizing esophageal stenosis. Cross-sectional imaging, although not routinely required, may provide additional information in selected cases, including exclusion of extrinsic compression and indirect clues regarding the underlying subtype, as observed in our patient, where imaging findings were suggestive of a fibromuscular form.

From a therapeutic perspective, our experience confirms that repeated and gradual endoscopic dilations represent an effective and safe first-line strategy in many cases of congenital esophageal stenosis. However, multiple sessions are often necessary to achieve sustained clinical improvement, and careful procedural planning is essential to minimize the risk of complications while optimizing outcomes.

The use of adjunctive therapies, such as topical budesonide, should be interpreted with caution. Although corticosteroids have shown potential benefit in reducing fibrosis and recurrence in acquired or post-procedural esophageal strictures, their role in congenital esophageal stenosis remains uncertain and is not supported by robust evidence [7,9]. In this context, their use should be considered empirical and individualized, rather than standard practice.

This case has several limitations. It represents a single-patient observation, and histological confirmation of the stenosis subtype was not available. The follow-up duration was relatively short, and long-term outcomes remain unknown. In addition, the independent contribution of topical budesonide to clinical improvement cannot be determined.

Overall, this case highlights the clinical relevance of early symptom recognition during weaning, the need for timely structural evaluation in infants with persistent or progressive symptoms, and the importance of a tailored, stepwise therapeutic approach. Early diagnosis is essential to avoid delayed treatment, unnecessary medical therapy, and feeding-related complications, ultimately improving clinical outcomes in affected patients.

## Data Availability

Data sharing is not applicable to this article as no datasets were generated or analyzed during the current study.
